# Radiographic signature in apical periodontitis improves prediction of apical lesion healing through survival prediction model

**DOI:** 10.1371/journal.pone.0327970

**Published:** 2025-07-21

**Authors:** Yuebo Liu, Ge Kong, Fantai Meng, Chunlan Guo, Kuo Wan

**Affiliations:** 1 Department of Stomatology, Peking Union Medical College Hospital, Peking Union Medical College, Chinese Academy of Medical Sciences, Beijing, China; 2 Ocean and Civil Engineering, School of Naval Architecture, Shanghai Jiao Tong University, Shanghai, China; Aga Khan University Hospital, PAKISTAN

## Abstract

This retrospective study aimed to evaluate the effectiveness of radiographic signatures of apical periodontitis (AP), particularly lesion boundary features, in predicting lesion healing periods using survival analysis. A total of 254 AP cases with apical lesions were included. Canny edge detection and fragment analysis (FA) were used to define the regions of interest (ROI) S1-S4 on radiographs. Radiographic signatures were extracted, and a radiomics score (rad-score) was developed using the least absolute shrinkage and selection operator (LASSO) Cox regression. Preliminary validation was performed using Kaplan-Meier survival analysis. Survival models were fitted, and model performance was evaluated. Clinical benefit was assessed through decision curve analysis. The results showed that radiographic signatures of the lesion boundary identified via the FA method significantly improved the performance of the survival model (Delong test; *p* < 0.05), with optimization of the calibration curve and an increase in the area under the curve (AUC) from 0.566–0.619 (reference model) to 0.884–0.905 at 12, 15, and 18 months. These findings were maintained in a small external validation cohort. The clinical benefit was also greater when using the rad-score derived via the FA method. In summary, the FA method proved to be an effective tool for quantifying the apical lesion boundary and predicting the healing speed using a survival model.

## Introduction

Apical periodontitis (AP) is a chronic inflammatory disease affecting the periapical tissues surrounding teeth with infected root canals. Radiographically, AP is typically characterized by radiolucency around the root of an affected tooth. In clinical practice, root canal therapy is the most common and effective treatment for AP. However, clinicians must often determine whether permanent restoration is appropriate or whether post-treatment disease persists—such as a residual apical lesion—necessitating additional intervention [1]. Therefore, monitoring the healing of apical lesions after root canal therapy, and tracking changes over time through periodic follow-up radiographic examinations, is essential.

Radiomics is a rapidly evolving field of research concerned with the extraction of quantitative metrics, the so-called radiomic features, from medical images. This trend is prominently shaping the fields of dentomaxillofacial radiology and endodontics, and an increasing number of studies have been dedicated to the development of models for clinical diagnosis and disease prognosis. Liang *et al.* proposed a new deep learning method (based on wavelet transform, an efficient image processing algorithm) for automatically diagnosing ameloblastoma, periapical cyst, and chronic suppurative osteomyelitis using cone-beam computerized tomography (CBCT) panoramic images; the model used only a small portion of labeled data and achieved 91.47% accuracy [2]. Wang *et al.* developed a neural network classifier to efficiently detect and diagnose periapical diseases from dental radiographs, enabling the precise segmentation and accurate classification of four periapical diseases: periapical granuloma, periapical abscess, periapical cyst, and condensing osteitis [3]. However, there is a relative paucity of radiomic studies focusing on the healing speed of AP with apical lesions on radiographs after root canal treatment.

In radiomics, texture analysis quantifies the spatial variation in pixel intensities within a region of interest (ROI), instead of a simple visual assessment. It helps to quantify tissue heterogeneity and can be applied to tasks such as lesion detection, tissue classification, and prognosis prediction. Many studies have investigated the application of texture analysis to characterize apical lesions of AP, and an association between the texture parameters derived from X-ray/CBCT images and histological diagnosis (e.g., radicular cysts and periapical granulomas) has been reported [4–6]. However, the current literature mainly focuses on the area of the apical lesion itself, and the boundary of the lesion may contain important diagnostic and prognostic information [7].

Based on the existing research, methods for quantifying apical lesion boundaries are relatively limited. The ROIs were manually annotated. Specifically, the first ROI encompassed the maximum area of the lesion interior and the second ROI encompassed an extension of this area to include the edge of the lesion. The texture parameters of the two ROIs were introduced separately into the model algorithm [7]. Previous research also applied Canny edge detection to quantify the lesion margin of parotid gland tumor and dental caries on medical images, thus achieving more objective results [8,9]. In this study, given that apical lesions are round, a new method was proposed for segmenting and reassembling radiographic images based on the mathematical concept of texture analysis (FA, fragment analysis) to improve the quantitative description of lesion boundaries.

In the current study, Canny edge detection and FA were employed to describe the apical lesion boundary of the AP, and texture parameters were extracted from the radiograph. A radiomics score (rad-score) was constructed based on the linear integration of radiographic signatures. Models based on survival analysis were fitted to the clinical data and rad-score. The model performance was evaluated using a validation cohort, and improvements in clinical utility were measured. This study aimed to investigate the effectiveness of radiographic signatures of apical lesions in AP, particularly at the lesion boundary, in predicting the lesion healing time using a prediction model, potentially influencing clinicians’ therapeutic decisions.

## Materials and methods

### Data source

#### Ethics and reporting guidelines.

This retrospective study was approved by the Ethics Committee of hospital (Peking Union Medical College Hospital). Informed consent was not required. Data were anonymized by removing all private radiographic tags and identifying information for each patient.

#### Patients.

Medical records from the institutional database were reviewed from January 1, 2021 to September 1, 2024 (accessed for research purposes on September 14, 2024). The inclusion and exclusion criteria were as follows.

The inclusion criteria were: (1) teeth diagnosed with AP, and apical lesions were confirmed by radiography; (2) apical lesion size between 5–15 mm in greatest diameter; (3) root canal treatment performed either by a single endodontist (LYB) or by others (GCL) following the same protocol as their routine treatment (details of the root canal treatment can be found in S1 Appendix); (4) patients were followed up with radiographs every 3 months postoperatively.

The exclusion criteria were: (1) teeth with periodontal disease; (2) teeth with severely compromised remaining tooth structure; (3) teeth requiring endodontic retreatment; (4) apical lesions on radiographs that overlapped with the maxillary sinus or other adjacent anatomical structures.

Computer-generated random numbers were used to assign patients to the development and validation cohorts in a ratio of 6: 4. The end point of this study was the complete healing of apical lesions on radiographs, which was confirmed separately by two board-certified dentists. Cohen’s kappa coefficient was used to measure the inter-rater reliability. Any disagreements were resolved by consensus with a third dentist after discussion. The healing criteria were based on the periapical index (PAI), and PAI-1 was used as the criterion for complete healing. Survival time was defined as the time from the date of radiographic examination at the initial visit to either the date of complete healing on radiography (event) or the date of the last known radiographic confirmation of the presence of an apical lesion (censored). The sample size was calculated at a confidence level (CI) of 95%. The healing rate was estimated based on previous studies [10,11]. A power test was conducted to evaluate the reliability of this study using the sample size and 4-year survival rates in both the development and validation cohort [12].

#### Clinical data.

Six basic clinical parameters, including gender, age, diabetes, lesion diameter on radiograph, tooth position (mandible or maxilla), and tooth location (anterior teeth, premolars, and molars), were collected. During or after data collection, the information of the individual participants was anonymous.

### Image acquisition and the determination of ROIs

#### Radiograph acquisition.

Periapical radiographs were obtained at the first visit (tube head, Sorede INTR Mlnray, Finland; plate scanner and sensor, DIGORA OPTIME, Finland). The exposure parameters for the anterior teeth, premolars, and molars were 70 kV, 7 mA, and 0.16 s for anteriors, 0.2 s for premolars, and 0.25 s for molars. A parallel technique was used.

#### Canny edge detection.

**Fig 1:**
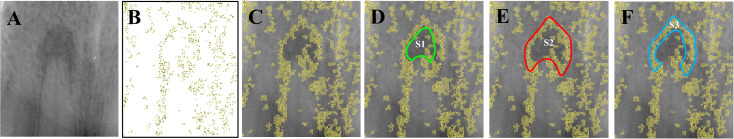
Representative example of the definition of ROIs S1, S2 and S3. Radiographic image of a typical apical lesion in maxillary anterior teeth (A). Canny edge detection was performed on radiographs (B). Overlaid image A and image B (C). S1 was defined comprising the maximum area of the lesion interior (D) and S2 comprising an extension of this area to include the edge of the lesion (E), while S3 was defined as the portion of S2 minus S1 (F).

The following procedure was applied to determine ROIs in the digital radiographic images (Fig 1): First, a Gaussian filter was applied to the image to remove noise. Subsequently, the intensity gradients of the images were identified. Subsequently, gradient magnitude thresholding or lower-bound cut-off suppression was applied to eliminate spurious responses to edge detection. Finally, a double threshold was applied to determine the potential edges, with the parameters set at 35/50 (lower threshold/higher threshold).

#### FA method: Segmenting and reassembling the boundary of lesion on radiograph.

**Fig 2:**
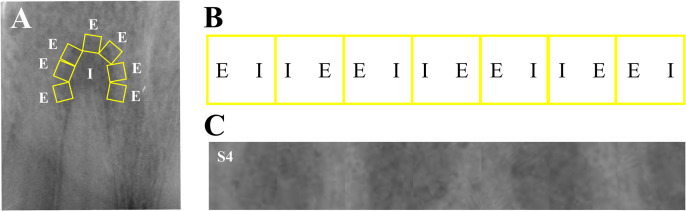
Representative example of the definition of ROIs S4. The grayscale gradient transition zone from lesion to surrounding tissue was totally covered with several squares (A). The radiograph was segmented according to the orientation of squares (B: I, the internal area of apical lesion; E, the external area of apical lesion). The segmented radiographic fragments were reorganized and reassembled, picture with light and dark vertical stripes was obtained (C).

The apical lesion on radiograph was processed according to the following rules: (1) the grayscale gradient transition zone from lesion to surrounding tissue was totally covered with several squares (1 mm × 1 mm); (2) the transition zone should be located in the center of the square and parallel to the side length; (3) squares cannot overlap but need to touch each other; (4) the radiograph was segmented according to the squares; (5) the segmented radiographic fragments were reorganized end to end in a certain order and reassembled picture with light and dark vertical stripes was obtained (Fig 2).

#### Determination of ROIs.

The subsequent procedure was executed based on the results of Canny edge detection. ROIs were manually delineated by a qualified oral radiologist (KG) using a semi-automatic active contour algorithm in MaZda software (version 4.6, Technical University of Lodz). ROI S1 contained the maximum area of the lesion interior; ROI S2 contained an extension of this area to include the edge of the lesion; and ROI S3 was defined as the portion of S2 minus S1. A reassembled image with light and dark vertical stripes was defined as ROI S4 (Figs 1 and 2).

### Construction and validation of rad-score

#### Rad-score building.

Texture parameters were extracted from all ROIs using MaZda software. The least absolute shrinkage and selection operator (LASSO) Cox regression model was used to identify the most important radiographic signatures in the development cohort. The rad-score was computed for each patient using a linear combination of the selected signatures weighted by their respective coefficients.

#### Validation of rad-score.

The potential association between the rad-score and periapical healing was assessed in the development cohort and subsequently validated in the validation cohort using Kaplan-Meier survival analysis. Patients were stratified into high-risk or low-risk categories according to the rad-score, with the threshold of this categorization determined using X-tile software (version 3.6.1; Yale University School of Medicine, New Haven, Conn) [13]. To ascertain the discrepancy in survival curves between the high- and low-risk groups, a weighted log-rank test (rho = 1, G-rho rank test) was performed. Subsequently, a univariate Cox regression analysis was performed, which yielded hazard ratios (HR) and 95% confidence intervals (CI).

### Model development and validation

#### Model established.

Model training was executed within the development cohort based on multivariate Cox analysis. Five models were fitted: a reference model based on six clinical data; model_1_: clinical data +S1 rad-score; model_2_: clinical data + S2 rad-score; model_3_: clinical data + S3 rad-score; model_4_: clinical data + S4 rad-score. A stepwise approach was applied during the variable selection. The internal validation of the regression analysis was performed using bootstrapping.

#### External validation and model performance.

External validation was performed using the validation cohort. The model calibration ability was evaluated using calibration curves, and model discrimination was measured based on time-dependent receiver operating characteristic (ROC) curves. The area under the curve (AUC) was calculated for each model, and the Delong test was used to compare the performance of the models based on their AUC values. Decision curve analysis (DCA) was performed to analyze the net benefit of the models in predicting the healing rate of the lesions.

### Statistical analysis

The R statistical software (version 3.6.1) was used for statistical analysis. The ‘pmsampsize’ R package was applied to perform sample size calculation and power test. Clinical variables were compared between the development and validation cohort using Pearson’s chi-square or Fisher’s exact test for categorical variables and the Mann-Whitney U test for continuous variables. The ‘foreign’ and ‘survival’ R packages were used to perform LASSO Cox regression model for rad-score building. Survival curves were obtained using the Kaplan-Meier method and compared using the log-rank test. Univariate and multivariate analyses were performed using Cox proportional hazard models. The ‘survminer’ and ‘time ROC’ packages were applied to calculate *p*-value that compared the AUC values, and ‘riskRegression’ package for calibration curve analysis between models. Finally, ‘dcurves’, ‘survival’ and ‘readr’ packages were applied for plotting DCA curves.

## Results

A total of 254 cases of AP with apical lesions confirmed on digital radiographs were included in this study. Of these, 152 and 102 cases were assigned to the development and validation cohorts, respectively (Table 1). In most cases, the tooth position was in the mandible, with the molars being the most common site. Until the last follow-up, 111 patients (73.03%) in the development cohort and 80 patients (78.43%) in the validation cohort had confirmed complete healing on radiography (median survival time was 18 months in both cohorts). No difference was found between the development cohort and the validation cohort in either clinical characteristics or follow-up data (*p* = 0.65–0.87). A power of 0.95 indicated that the sample size for development and validation in this study was sufficient. The kappa value was 0.753 for the case inclusion process, indicating a substantial agreement between the two dentists.

**Table 1:** Participant characteristics in development cohort and validation cohort.

Characteristics	Development cohort (152)	Validation cohort (102)
Gender (female)	56	41
Age (years)^a^	52.89 ± 19.74	50.60 ± 18.49
Diabetes	46	36
Lesion size (mm)^a^	10.12 ± 3.34	9.71 ± 3.77
Tooth position		
Maxilla	53	34
Mandible	99	68
Tooth location		
Anterior teeth	43	33
Premolars	42	27
Molars	67	42
Follow up time (month)^b^	18 (15, 27)	18 (12, 21)

Unless otherwise specified, data are numbers of patients. No difference was found between the development cohort and the validation cohort in either the clinical characteristics or the follow-up data (*p* = 0.65–0.87).

^a^ Numbers in this item are present as mean ± standard deviation.

^b^ Numbers in this item are present as median (first quartile, third quartile).

**Fig 3:**
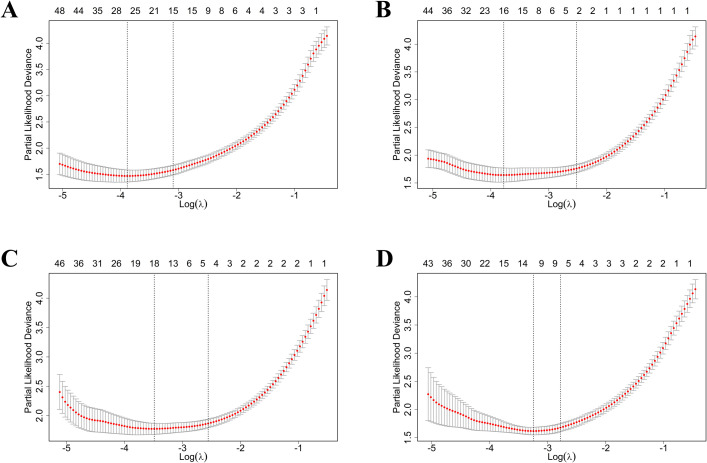
Radiomic feature selection using LASSO regression model. Radiomic feature in S1 (A), S2 (B), S3 (C) and S4 (D) were present.

In the LASSO Cox model (model_1–4_), *λ* values of 0.045, 0.080, 0.077, and 0.062 were selected, respectively, using a tenfold cross-validation approach to identify the optimal signatures. As a result, 15 features were selected as signatures in model_1_, three features in model_2_, five features in model_3_, and five features in model_4_, with nonzero coefficients (Fig 3). A complete list of radiographic signatures and their respective coefficients can be found in the S2 Appendix. Finally, the rad-score was computed for each patient using a linear combination of the selected features, weighted by their respective coefficients.

**Fig 4:**
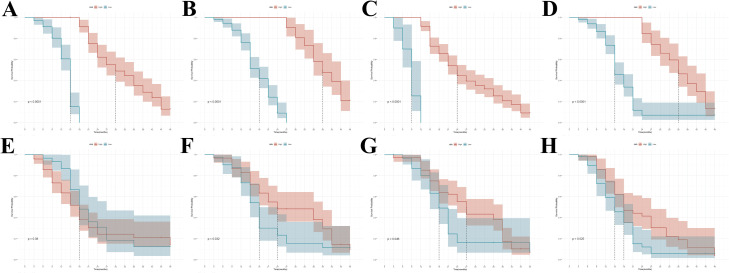
Kaplan-Meier curves analysis based on rad-score. Graphs showed results of Kaplan-Meier survival analyses according to the rad-score for patients in the development cohort (A, B, C and D) and those in the validation cohort (E, F, G and H). Kaplan-Meier curves in S1 (A and E), S2 (B and F), S3 (C ang G) and S4 (D and H) were present. A significant association of the rad-score with the apical lesion healing was shown in the development cohort (*p* < 0.05), which was then confirmed in the validation cohort except the rad-score of S1 (*p* = 0.38). Colored shaded areas = two-sided CI of the survival curves (blue line: low risk group; red line: high risk group).

In the development cohort, the rad-score thresholds for classifying patients into high/low-risk groups were 2.1, 1.9, 3.9 and 0.9 in model_1–4_, which were identified using the X-tile. The results showed that the rad-score was able to distinguish between the two Kaplan–Meier curves. In the validation cohort, the rad-score was associated with the healing of apical lesion in model_2_ (*p* = 0.046; HR = 1.573, CI: 1.051, 2.421), model_3_ (*p* = 0.032; HR = 1.632, CI: 1.057, 2.572) and model_4_ (*p* = 0.025; HR = 1.663, CI: 1.063, 2.603). However, this correlation was not found in model_1_ (*p* = 0.38; HR = 1.177, CI: 0.731, 1.897), and was discarded in the following analysis. The results indicated that patients with higher rad-score generally had shorter healing periods for apical lesions (Fig 4).

**Fig 5:**
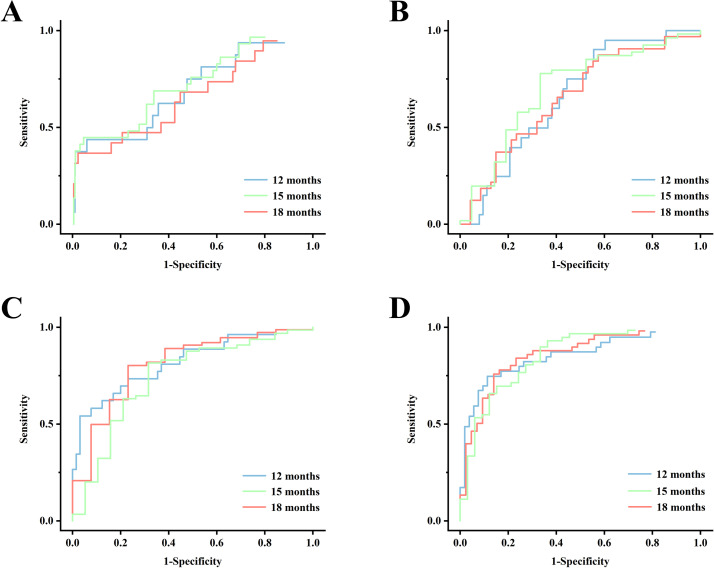
Receiver-operating characteristic (ROC) curves of reference model (A), model_2_ (B), model_3_ (C) and model_4_ (D) in the validation cohort.

**Fig 6:**
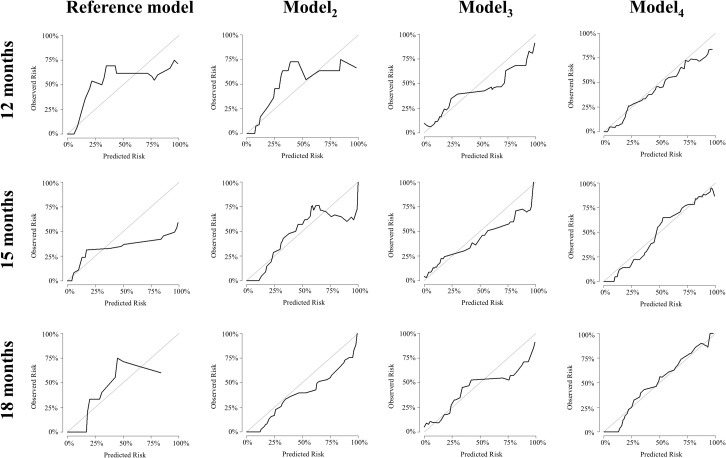
Calibration curves of the Cox models at the time point of 12, 15 and 18 months in validation cohort. Calibration curves depict the calibration of each model in terms of the agreement between the predicted risks of predicted overall survival and actual overall survival. The *y*-axis represents the observed risk. The *x*-axis represents the predicted risk. The line (*y* = *x*) represents a perfect prediction by an ideal model.

To compare the ability of the models to predict the healing of apical lesions, their discrimination and calibration abilities were measured. For instance, at 15 months, the AUC and Delong test revealed a substantial enhancement in model discrimination (Table 2). The AUC increased from 0.621 in the reference model to 0.701, 0.779, and 0.892 in model_2_, model_3_, and model_4_, respectively, in the external validation cohort (*p* < 0.05, in the Delong test). Additionally, an enhancement in calibration was evident in model_2_ (calibration slope, 0.819), model_3_ (calibration slope, 0.861), and model_4_ (calibration slope, 0.909) compared to the reference model (calibration slope, 0.685) (Table 3). The details of the ROC and calibration curves are presented in Figs 5 and 6, respectively.

**Table 2:** Discrimination performance of predictive models in validation cohort.

	AUC [95%CI]	Delong test			
		Reference Model	Model_2_	Model_3_	Model_4_
Reference Model					
12 months	0.619 [0.587-0.651]	–	–	–	–
15 months	0.621 [0.538-0.704]	–	–	–	–
18 months	0.566 [0.437-0.694]	–	–	–	–
Model_2_^*^					
12 months	0.688 [0.587-0.789]	**−2.64**	–	–	–
15 months	0.701 [0.594-0.808]	**−2.68**	–	–	–
18 months	0.675 [0.533-0.817]	**−2.79**	–	–	–
Model_3_^*^					
12 months	0.801 [0.736-0.867]	**−4.67**	**−2.66**	–	–
15 months	0.779 [0.661-0.898]	**−4.21**	**−2.38**	–	–
18 months	0.802 [0.697-0.907]	**−4.82**	**−2.73**	–	–
Model_4_^*^					
12 months	0.905 [0.864-0.946]	**−7.32**	**−5.02**	**−2.95**	–
15 months	0.892 [0.811-0.973]	**−7.42**	**−4.53**	**−2.87**	–
18 months	0.884 [0.799-0.969]	**−7.21**	**−4.18**	**−2.85**	–

Abbreviations: AUC, area under the curve; 95%CI, 95% confidence interval.

* Bold number indicates significant difference (*p* < 0.05).

**Table 3:** Calibration performance of predictive models in validation cohort.

	Calibration slope	Intercept
Reference Model		
12 months	0.734	0.312
15 months	0.685	0.229
18 months	0.712	0.351
Model_2_		
12 months	0.831	0.316
15 months	0.819	0.267
18 months	0.824	0.294
Model_3_		
12 months	0.856	0.233
15 months	0.861	0.295
18 months	0.872	0.197
Model_4_		
12 months	0.912	0.192
15 months	0.909	0.183
18 months	0.923	0.205

**Fig 7:**
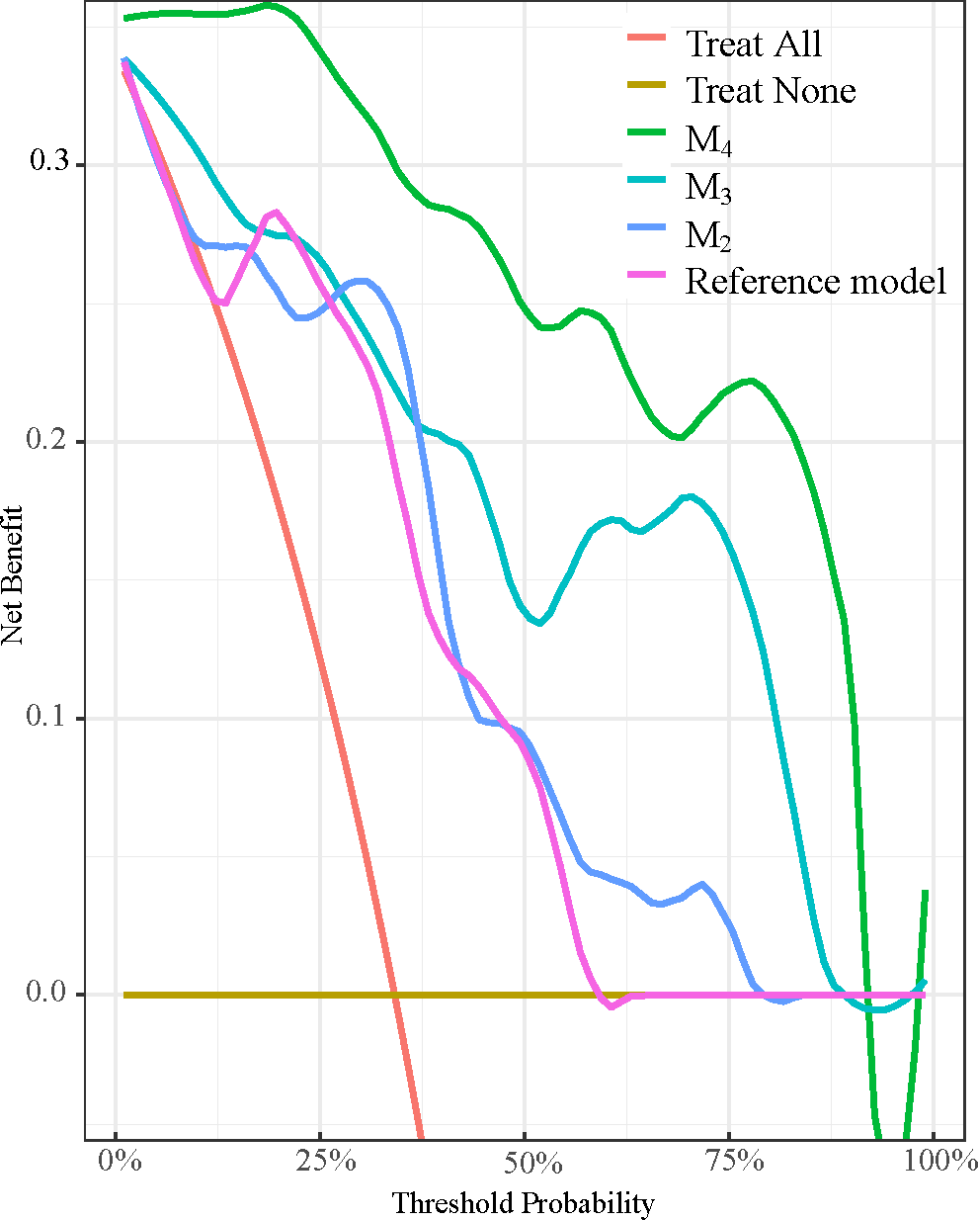
Decision curves of reference model, model_2_, model_3_ and model_4_ in predicting the periapical healing. The *y*-axis measures the net benefit. The red line represents the net benefit of predicting all patients suffering from long-term periapical healing, and the brown line represents all patients are predicted healing immediately.

The decision curve analysis demonstrated that, in comparison with model_3_ and model_4_, the reference model and model_2_ exhibited a high degree of similarity in their respective curves, irrespective of the selected threshold. The clinical application of model_4_ was found to yield benefits that were significantly higher than those of model_3_ (Fig 7).

## Discussion

In clinical practice, teeth with apical lesions require periodic follow-up via radiographic examination after root canal therapy. Predicting the healing time of apical lesions is essential when deciding whether to perform permanent restoration, or periapical surgical intervention. However, evaluating the healing rate of apical lesions is challenging, particularly for dentists lacking sufficient experience. In addition, only a limited number of studies are available in this field. With the development of radiomics, image mining toward clinical applications has attracted more attention [14]. In this study, radiomics was used as a valuable tool for analyzing apical lesion boundaries, with the objective of developing a model to predict apical lesion healing. This study was designed to assist clinicians in analyzing dental radiographs more effectively, thereby facilitating informed therapeutic decision-making.

The boundary of apical lesions on radiographs has diagnostic value in clinical practice [7]. Traditionally, the description of borders can be subcategorized as sharp demarcation, corticated borders and sclerotic borders, which are related to pathological classification [15]. However, evaluation of lesion borders primarily depends on the clinician’s experience. In medical imaging, texture analysis is a method for measuring variations in pixel intensities across a given image, ROI, or volume. This variation is often imperceptible or challenging to quantify by human perception. Images with rough textures exhibit a high degree of change in pixel intensity between high and low values, in contrast to images with smooth textures. Previous studies have successfully quantified the grayscale gradient transition zone of caries on radiographs [9], and the boundaries of parotid gland tumors on magnetic resonance imaging (MRI) [8] via texture analysis. However, many texture features, for example, such as those based on Gray Level Co-occurrence Matrix (GLCM) and Gray Level Run-Length Matrix (GLRLM), can only quantify the pattern of gray level intensity pixels in a fixed direction from an interference pixel [16]. Therefore, they are sensitive only to the texture patterns in certain directions. For apical lesions with circular boundaries, a new method of segmenting and reassembling radiographic images was developed to improve texture analysis results in the current study. To the best of our knowledge, this is the first study to report the aforementioned method for establishing a predictive model for periapical healing. This novel approach enabled the straightforward and intuitive interpretation of specific model coefficients. For example, the GLCM contrast quantifies the intensity contrast between an index pixel and its surrounding neighborhood pixels. A higher contrast generally indicates deeper grooves in the texture and clearer visual effects. Results showed that S (5, 0) contrast was a risk factor for periapical healing and might be interpreted as follows: a vague and thicker border is indicative of a lesion with acute nature [17], thus indicating that the periapical bone has not totally broken down, and less time is needed for hard tissue regeneration [18]; Besides, a corticated border is commonly observed with cysts, and some of cysts especially true cyst, because of its self-sustaining nature, is less possible to improve after non-surgical root canal treatment [19].

Before studying the predictive value of lesion boundaries using different radiographic segmentation methods, the influence of clinical factors must be ruled out. A previous systematic study suggested a strong connection between the presence of periapical radiolucency in root-filled teeth among diabetics and that diabetes may affect the healing and survival of root-filled teeth with apical lesions [20]. In the current study, diabetes mellitus was included as one of six basic clinical parameters. In addition, there was a significant difference in the outcomes among the anterior, premolar, and molar teeth. A more favorable outcome was observed in roots with one canal than in those with two or more canals because of the challenge of disinfecting complex canal systems [21]. Therefore, tooth position was included as a factor. Besides, age and bone loss diameter were also considered in multivariate survival analysis [11,22]. In the inclusion criteria, the size of apical lesion was limited to 5–15 mm. Lesions smaller than 5 mm are not suitable for the FA method, and lesions larger than 15 mm may have a longer healing period, which reduces the incidence of events and increases the censored data in the survival analysis [11].

According to Harrell’s guidelines, the number of events should exceed the number of included covariates by at least 10 times in multivariate analysis [23]. LASSO is a statistical formula suitable for the regression of high-dimensional data, and its main purpose is feature selection and the regularization of data models. The LASSO regression is ideal for predictive problems, and its ability to perform automatic variable selection can simplify models and enhance prediction accuracy by reducing the coefficients towards zero. These variables could be any combination of continuous, binary, or categorical data. In survival analysis, LASSO Cox regression model can also be used to select the most useful prognostic features in the development cohort. Previous studies indicated that the LASSO technique for variable selection combined with the Cox model was less variable than the stepwise approach and yielded more interpretable models [24,25].

Calibration assessment is a pivotal element in the derivation and validation of clinical prediction models. Calibration is the degree of concordance between predicted and observed risks. In contrast to binary outcomes, the calibration of models for time-to-event outcomes entails evaluating the agreement between the observed and estimated probabilities of an event transpiring within a stipulated duration. Thus, calibration in this setting is assessing observed and predicted probabilities at a specific time point [26]. Carolina *et al.* reported that more than 60% of patients with apical lesions displayed periapical bone healing between months 12 and 18 on cone beam computed tomography [11]. Therefore, 12, 15, and 18 months were selected for the calibration assessment in this study. Results indicated that the predicted risk of the model tended to be overestimated at 12 months, and underestimated at 18months. This result needs to be interpreted in light of the clinical reality. In clinical practice, the placement of a crown after endodontic treatment depends on various factors. Previous studies have shown that the functional loading of a full-coverage prosthesis in endodontically treated mandibular molars with pulp necrosis and asymptomatic apical periodontitis delays periapical healing [27]. In some circumstances, the occlusal trauma on full-coverage crown may also be one of the factors responsible for endodontic failure [19,28,29]. However, if crown placement is delayed and the tooth is subjected to excessive force or direct trauma, a subsequent fracture may occur. Therefore, a decision must be made on whether to follow up until full bone healing or to obtain a final restoration immediately to reduce the likelihood of a crown fracture. In general, a longer periapical healing period is usually associated with larger or more severe infection of the lesion and a relatively weaker repair capacity of the host’s immune response [30]. Full bone healing without the interference of occlusal force should be the most important issue when making clinical decisions. In contrast, for teeth with shorter periapical healing times, placing the crown as soon as possible may improve the long-term success rate of root canal therapy. Therefore, a slight overestimation at 12 months and underestimation at 18 months may be acceptable.

In recent years, the advent of non-invasive techniques for lesion characterization has led to the emergence of MRI as a promising method for the analysis of apical lesions. A key benefit of MRI over conventional methods such as CBCT and radiography is its superior soft tissue contrast. Additionally, the flexibility to modulate contrast through the design of MRI sequences further enhances diagnostic capabilities. The study showed that the texture parameters of the apical lesion center, which were discarded owing to their poor performance in the current study, were related to the pathological characteristics of MRI images [31]. Therefore, multiple detection methods should be combined in future studies to obtain more reliable results.

Current study has some limitations. First, it should be noted that similarly well-defined lesion generally appears less defined in the maxilla than in the mandible, particularly in plain 2-Dimensional views, which are related to the trabecular architecture and also the thickness of the bone [15]. Therefore, the measurement and analysis of CBCT should be considered to obtain more accurate and repeatable texture parameters [11]. Second, in future studies, Canny edge detection and the FA method can be software-integrated instead of being manually operated, thus reducing operational errors and improving the reliability of the results. Third, in future studies, the use of propensity score matching to balance groups when adjusting for potentially unknown confounding factors is recommended. Finally, the sample size was not large, and multi-center validation with a larger sample size is essential to acquire a robust analysis for future clinical applications.

## Conclusion

The efficacy of FA in analyzing the apical lesion boundaries of the AP has been demonstrated. The incorporation of radiographic signatures such as the S (5, 0) contrast obtained by texture analysis has been shown to enhance the performance of the model. These findings were validated using a small external cohort. Furthermore, the application of radiographic signatures has been demonstrated to provide enhanced net benefits in development and validation cohorts, underscoring their potential role as supplementary tools for predicting the healing of apical lesions. However, further validation is necessary through studies employing multicenter cohorts to confirm these conclusions.

## Supporting information

S1 Appendix Clinical procedure. (DOCX)

S2 Appendix Details of model coefficients. (DOCX)
