# Supplementary material for: Radiographic signature in apical periodontitis improves prediction of apical lesion healing through survival prediction model
Source: PLoS One. 2025 Jul 21;20(7):e0327970. doi: 10.1371/journal.pone.0327970 (PMC12279126; doi:10.1371/journal.pone.0327970)
Supplement: S1 Appendix — (DOCX) [file pone.0327970.s001.docx]

**S1 Appendix**

**Clinical procedure**

Root canal treatment was performed under rubber dam isolation with the application of a magnifying device. An adequate glide path was established with manual files C file (Dentsply Maillefer, USA), followed by the rotary instrumentation system (Mtwo file system, VDW, Germany). A generous amount of 1% sodium hypochlorite was used with ultrasonic activation during mechanical instrumentation. Intracanal medication (Endo-calcium, Spain) was applied between visits. Finally, the canal system was filled using the single-cone technique together with AH Plus sealer (Dentsply, Germany). Fuji IX GP (GC, Japan) was used for coronal filling after root canal treatment until the coronal restoration was placed.
