# Supplementary material for: Radiographic signature in apical periodontitis improves prediction of apical lesion healing through survival prediction model
Source: PLoS One. 2025 Jul 21;20(7):e0327970. doi: 10.1371/journal.pone.0327970 (PMC12279126; doi:10.1371/journal.pone.0327970)
Supplement: S2 Appendix — (DOCX) [file pone.0327970.s002.docx]

**S2 Appendix**

**Details of model coefficients**

**Model_1_**

Skewness -0.01283634

Perc.10. -0.03118874

Perc.50. -0.07964382

Perc.90. -0.46126541

Perc.99. -0.22538788

S (1, 0) DifVarnc -1.93162364

S (0, 1) InvDfMom -0.47264553

S (0, 1) SumAverg -0.43424470

S (1, -1) Entropy -0.91869403

S (0, 2) InvDfMom -0.04859231

S (5, 0) AngScMom 0.11512510

Vertl GLevNonU 0.07094060

Sigma 0.05210499

WavEnLL s-6 -0.02773146

WavEnHH s-6 0.04825284

**Model_2_**

S (5, -5) Entropy 3.2442144131

S (5, 0) contrast -0.0286554887

GrVariance -0.0008499089

**Model_3_**

Perc.01. -1.117886884

S (5, 0) contrast -1.907877164

S (0, 1) Correlat 0.005560241

S (0, 1) DifEntrp -0.038310929

WavEnHH s-2 0.197591226

**Model_4_**

S (1, 0) DifEntrp 0.12852025

S (5, 5) Entropy 1.51976611

Vertl LngREmph 0.09473661

S (5, 0) contrast -2.05355750

X135dr GLevNonU -0.02180278
